# Inflammation and Nutrition: Friend or Foe?

**DOI:** 10.3390/nu15051159

**Published:** 2023-02-25

**Authors:** Franziska Stumpf, Bettina Keller, Carla Gressies, Philipp Schuetz

**Affiliations:** 1 Medical University Department of Medicine, Division of General Internal and Emergency Medicine, Kantonsspital Aarau, 5001 Aarau, Switzerland; 2 Department of Nutritional Medicine and Prevention, Institute of Clinical Nutrition, University of Hohenheim, 70599 Stuttgart, Germany; 3 Medical Faculty, University of Basel, 4056 Basel, Switzerland

**Keywords:** malnutrition, screening, inflammation, nutritional support, clinical outcomes, precision medicine, personalized nutrition

## Abstract

The importance of the interplay between inflammation and nutrition has generated much interest in recent times. Inflammation has been identified as a key driver for disease-related malnutrition, leading to anorexia, reduced food intake, muscle catabolism, and insulin resistance, which are stimulating a catabolic state. Interesting recent data suggest that inflammation also modulates the response to nutritional treatment. Studies have demonstrated that patients with high inflammation show no response to nutritional interventions, while patients with lower levels of inflammation do. This may explain the contradictory results of nutritional trials to date. Several studies of heterogeneous patient populations, or in the critically ill or advanced cancer patients, have not found significant benefits on clinical outcome. Vice versa, several dietary patterns and nutrients with pro- or anti-inflammatory properties have been identified, demonstrating that nutrition influences inflammation. Within this review, we summarize and discuss recent advances in both the role of inflammation in malnutrition and the effect of nutrition on inflammation.

## 1. Introduction

Disease-related malnutrition (DRM) is a common syndrome in patients with acute and chronic illnesses. Prevalence rates are approximately 30% among medical inpatients and rise higher among the elderly or critically ill [1,2]. Left untreated, DRM is associated with poor outcomes, such as higher mortality and prolonged intensive care unit (ICU) and hospital stays [3,4]. Inflammation, undernutrition-driven catabolism, and inadequate dietary intake are key drivers of DRM [1]. While medical inpatients with malnutrition have been shown to benefit from nutritional treatment, this may not be equally true for other patient populations, such as those in the ICU [1,5]. A Cochrane review from 2017 on a more heterogenic patient population, which included highly inflamed patients such as those in the ICU, only found limited positive effects of nutritional treatment on clinical outcomes [6].

The focus on inflammation as a key driver of DRM has grown due to the growing understanding of DRM and its pathophysiology. Recent studies have shown associations between inflammatory processes measured by inflammation biomarkers, such as C-reactive protein (CRP), and responses to nutritional therapy [7]. The many possible reasons will be discussed within the scope of this review, in conjunction with the pathophysiological connection of inflammatory responses in illness and DRM. As researchers strive to elucidate the role of inflammation in nutrition, the interest in potential anti-inflammatory properties of nutrition also grows. We aim to provide an overview of the influence of nutrition on inflammation through a discussion of dietary patterns such as the Mediterranean diet (MD), and various indices such as the Dietary Inflammatory Index (DII) and nutrients (ex. fatty acids [FA]).

## 2. Malnutrition and Inflammation

DRM is a complex syndrome resulting from the inadequate intake of nutrients required to support physiological function and disease-related systemic inflammatory responses. The European Society of Clinical Nutrition and Metabolism (ESPEN) defines malnutrition as “a state resulting from lack of intake or uptake of nutrition that leads to altered body composition (decreased fat free mass) and body cell mass leading to diminished physical and mental function and impaired clinical outcome from disease” [8]. There remains considerable scope for confusion and misunderstanding; however, this is only one of several definitions found in the literature.

In hospitalized patients, malnutrition is not only due to inadequate nutritional intake, but is often disease-related and associated with complex pathophysiological mechanisms. These mechanisms can differ depending on the underlying disease and its treatment. Inadequate dietary intake has also been shown to lead to immune system dysfunction and mucosal damage in the gut [9]. In the presence of DRM, hospital food, gastrointestinal symptoms, and dysfunction can contribute to reduced appetite, food intake, and nutrient assimilation. The lack of movement can further contribute to malnutrition [1].

### 2.1. Malnutrition—Risk Factors and Diagnosis

DRM is multifactorial: risk factors include polypharmacy, disease-related inflammatory mechanisms, compromised intake or assimilation of nutrients, immobility associated muscle wasting, older age, and social isolation [1,10]. In addition to an already high prevalence of malnutrition upon admission, nutritional states may be further aggravated during hospitalization due to illness-related loss of appetite, fasting for diagnostic tests, drug-induced side effects, diseases that affect gastrointestinal function, or other factors associated with hospitalization [1]. To diagnose malnutrition, the Global Leadership Initiative on Malnutrition (GLIM) proposes a two-step approach consisting of nutritional risk screening followed by a more thorough evaluation. There is no one universal screening method for malnutrition but rather a number of different tools which have been validated for different settings, including the NRS-2002 [11], SGA [12], MUST [13] or MNA-SF [14]. If nutritional risk is identified, a nutritional assessment to confirm a diagnosis should be performed, including etiological (reduced food intake or assimilation and disease burden/inflammation) and phenotypic (non-volitional weight loss, low BMI and reduced muscle mass) criterions. According to GLIM, a diagnosis of malnutrition is fulfilled if one etiological and one phenotypic criterion apply for the patient [15].

### 2.2. Malnutrition—Classification

**Figure 1:**
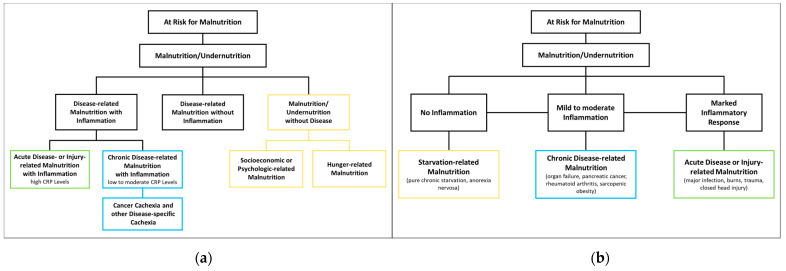
Classification of Malnutrition by (**a**) European Society of Clinical Nutrition and Metabolism (ESPEN) [8] and (**b**) American Society for Parenteral and Enteral Nutrition (ASPEN) [16].

The ESPEN proposes three etiological groups: DRM with inflammation, DRM without inflammation, and malnutrition/undernutrition without disease (Figure 1a). DRM with inflammation can be divided further into acute and chronic forms. Chronically malnourished patient groups typically affected by inflammation (and thus cachexia) include patients with cancer, chronic obstructive pulmonary disease (COPD), inflammatory bowel diseases, congestive heart failure, chronic kidney disease, and other end-stage organ diseases. Inflammation in these patients is often milder, with CRP levels of up to 40 mg/l. DRM with inflammation due to acute disease or injury typically affects critically ill patients or post major surgery, and is accompanied by higher levels of CRP [8]. The American Society for Parenteral and Enteral Nutrition (ASPEN) uses a similar approach, categorizing according to (I) social and environmental circumstances, (II) chronic illness and (III) acute illness or injury (Figure 1b) [16]. They also specify that malnutrition caused by social and environmental circumstances is not related to disease and corresponds to “malnutrition without disease”. As the latter is not relevant in the context of disease, it will not be discussed in this review.

### 2.3. Malnutrition—Therapy and Clinical Outcomes

Malnutrition has been shown to be a risk factor for adverse outcomes such as increased mortality, a higher risk of readmission within 30 days, prolonged hospital and ICU stays, loss of function, and infection rates [2,3,4]. While the possible benefits of nutritional therapy in malnourished patients have long been unclear [6,17], recent evidence in favor of applying nutritional therapy in medical inpatient settings has been growing, in part due to large-scale RCTs including the EFFORT [18] and the NOURISH trials [19]. In a recent meta-analysis, Gomes et al. found that nutritional therapy significantly improves functional outcome and reduces loss of body weight, risk of complications, and hospital readmission. Recent trials included in the meta-analysis have indicated a marked decrease in risk of mortality compared to older studies (OR 0.47, 95% CI 0.28–0.79) [20]. In addition, trials with higher protein intake and longer intervention times have been associated with a stronger impact of nutritional support on clinical outcomes [21]. All the above-mentioned reasons make early recognition and adequate treatment of malnutrition vital for each patient’s individual treatment plan.

### 2.4. Inflammation in Malnutrition

**Figure 2:**
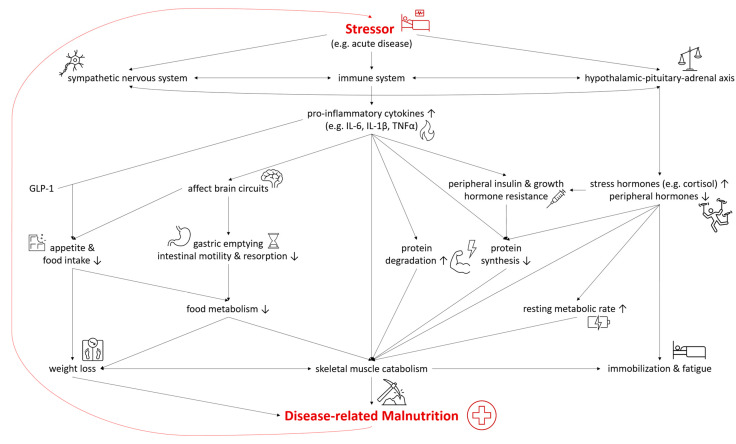
Selected Pathways in the Interplay of Inflammation and the Pathophysiology of Disease-related Malnutrition. IL-6, interleukin 6; IL-1β, interleukin 1β; TNF-α, tumor necrosis factor α; GLP-1, glucagon-like Peptide-1.

In both DRM with acute and chronic inflammation, the sympathetic nervous system, the immune system, and the hypothalamic–pituitary–adrenal axis are activated as a systemic response to a stressor and disease [22,23]. As they are connected both anatomically and functionally, they interact in the response to the stressor [24]. The modulation of the hypothalamic–pituitary–adrenal axis stimulates the release of stress hormones, including cortisol, catecholamines, and suppresses other hormones regulating sex, thyroid, and other peripheral functions [22]. In malnutrition, the deiodination of thyroxine (T4) to triiodothyronine (T3) was shown to be down regulated—a process called “low T3 syndrome” which is an adaptive metabolic mechanism to reduce energy expenditure and prevent catabolism [25]. Catecholamines and cortisol increase glycogenolysis and gluconeogenesis in the liver while simultaneously inducing peripheral insulin resistance and inhibiting glucose from entering cells [22]. Furthermore, insulin-dependent glucose transporters in peripheral tissues are downregulated, causing stress hyperglycemia [26]. Pro-inflammatory cytokines including interleukin 6 (IL-6), interleukin 1β (IL-1β) and tumor necrosis factor α (TNF-α) are released, triggering several mechanisms which contribute to the pathogenesis of malnutrition (Figure 2). Pro-inflammatory cytokines also affect brain circuits which control food intake, cause delayed gastric emptying and increase skeletal muscle catabolism [1,23,27,28]. Furthermore, researchers have identified an interaction of pro-inflammatory cytokines (mainly IL-6 and IL-1β) and gut tissue-released glucagon-like peptide-1 (GLP-1), resulting in reduced food intake and unintentional weight loss [29]. Muscle degradation is triggered by decreased synthesis of muscle protein and the increased degradation of proteins such as myosin heavy chains [9]. These endocrine changes further advance catabolism and lead to fatigue and immobilization [1,23]. The combination of these mechanisms leads to compromised food metabolism, a hypercatabolic state, and eventually to DRM.

## 3. Is Nutrition a Friend? How Nutrition influences Inflammation

Primarily, nutrition serves as the source of essential nutrients, providing energy and substrates for the numerous metabolic functions. However, as nutrition has long been a topic of scientific interest, knowledge about specific properties of nutrition or nutrients has grown with its anti-inflammatory properties gaining much attention [30]. While inflammation is an acute reaction to stressors such as trauma or infection, an increasing number of chronic diseases such as cardiovascular diseases, vascular cognitive impairment, and dementia have also been associated with chronic (often subclinical) inflammation [31].

### 3.1. Anti-Inflammatory Potential of Nutrients and Other Food Components

Numerous food components have been investigated for their anti-inflammatory properties and potential use in nutritional therapy. Research has focused not only on macronutrients such as fatty acids or amino acids and micronutrients such as vitamin D, but also included other food components such as fiber and phytochemicals [32,33,34,35]. A selection of these is described in more detail below.

#### 3.1.1. Omega-3 and Omega-6 Fatty Acids

Polyunsaturated fatty acids (PUFA), especially omega-3 (n-3 FA) and omega-6 fatty acids (n-6 FA), are amongst the most studied macronutrients in this context. As the human body is not able to synthesize these essential elements, they must be ingested from an outside source. Long-chain n-3 FA (LC n-3 FA) such as eicosapentaenoic acid (EPA), docosapentaenoic acid (DPA), or docosahexaenoic acid (DHA) are found in aquatic organisms or can be metabolized from plant-derived α-linolenic acid (ALA) [36]. While n-6 FA linolenic acid (LA) is commonly found in vegetable oils such as sunflower oil, long-chain n-6 FA (LC n-6 FA), arachidonic acid (AA) is found in animal products such as meat or egg or is biosynthesized from LA. LC n-3 FA and LC n-6 FA are then used as substrates for mediators such as prostaglandins, thromboxanes, and leukotrienes. While these n-6 FA products are considered pro-inflammatory, products within the n-3 FA pathway are considered anti-inflammatory. Due to the role of the same enzymes in both pathways, n-3 FA possesses the potential to competitively reduce the metabolism of n-6 FA to pro-inflammatory mediators. However, results on the pro-inflammatory effect of n-6 FA are conflicting, as some studies did not find a significant association with inflammation biomarkers or even reported anti-inflammatory effects [37,38]. This is also reflected in the literature-based DII (described in detail below) which calculated an anti-inflammatory potential for n-6 FA [39]. The effects of n-3 and n-6 FA have been extensively studied in relation to cardiovascular diseases [40], as well as other chronic illnesses associated with inflammation such as rheumatoid arthritis [41] or cancer cachexia [42,43].

A growing number of studies on various cancer entities show n-3 FA supplementation to have a positive effect, e.g., reduced inflammatory markers and increased fat, skeletal muscle, and lean body mass [42,43]. Despite the physiological rationale and considerable number of high-quality trials on the beneficial effects of n-3 FA in cancer patients, the amount of evidence remains low to moderate overall, with the ESPEN guideline on clinical nutrition in cancer only publishing a weak recommendation for n-3 FA usage [42]. Similarly, two recent Cochrane reviews on the preventive effects of n-3-FA and n-6-FA on cardiovascular disease [32,33] found only low to moderate certainty evidence for their respective effects. They did however conclude that n-3 FA supplementation may reduce the risk of coronary heart disease, mortality, and cardiovascular events, and lead to a reduction in serum triglycerides. Additionally, substitution of saturated FA with n-6 FA may decrease the rate of myocardial infarctions and lead to a lower serum cholesterol [32,33].

#### 3.1.2. Saturated and Trans-Fatty Acids

In contrast to the n-3 FA, other FA such as trans-FA seem to have predominantly pro-inflammatory properties. In “Western” diets, the main source of trans-FA are partially hydrogenated oils, usually the result of industrial processing, and partly derived by microbial processes in ruminant animal products [44]. They lead to cell toxicity through increased oxidative stress, increased radical oxygen species (ROS) production, or damage of the endoplasmic reticulum. Furthermore, incorporation of trans-FA into components of the cell membrane may lead to modulation of cellular signaling pathways related to inflammation. In contrast, the effects of saturated FA on inflammation are not yet clear [30]. Most studies exploring their effect on inflammation focus on entire meals high in saturated FA rather than on the individual FAs. Current evidence suggests that LC-saturated FA exert a pro-inflammatory effect due to an increased production of ROS and an activation of pro-inflammatory pathways. Short- and medium-chain saturated FA on the other hand seem to have no such effect, and may potentially possess anti-inflammatory properties [30].

#### 3.1.3. Carbohydrates and Fiber

Fiber is another nutritional component, known to have anti-inflammatory properties [45]. Fiber-rich diets are often associated with a high intake of polyphenols and complex carbohydrates, both of which may affect inflammation positively. One anti-inflammatory mechanism of fiber is due to the conversion of non-digestible carbohydrates into immune-regulating substances (ex. short-chain FA [SCFA]) by the gut microbiota. These SCFA are converted into acetyl-CoA, which can activate signaling pathways via G protein-coupled receptors. Activation can promote gene transcription in the nucleus by inhibiting histone deacetylases and is followed by activation of the peroxisomal proliferator-activated receptor gamma (PPAR-γ), and inhibition of nuclear factor-kappa B (NF-κB) activation. This decreases the inflammatory response by reducing cytokine, TNF-α, MCP-1 or IL-6 production [30]. They may also increase the intrinsic availability of antioxidant substances such as vitamins or carotenoids by carrying them into the gastrointestinal tract where they help to maintain a normal intestinal flora. Furthermore, foods containing complex carbohydrates and fiber have been reported to reduce low-density lipoprotein (VDL) as well as inflammatory markers such as CRP, plasminogen activator inhibitor, Il-6 or TNF-α [46]. In diabetic patients, for instance, an increase in fiber consumption resulted in decreased CRP levels [30,47].

Products with high levels of free added sugar, on the other hand, seem to have enhanced pro-inflammatory effects. An increase in blood glucose caused by sugar-rich food can lead to the formation of advanced glycation end products (AGE), caused by non-enzymatic interaction between glucose and amino acids, proteins, or peptides. Some AGE (termed toxic AGE) may cause oxidative stress, trigger inflammatory processes, or induce cell death. Several possible mechanisms of inflammation have been identified, including the binding of AGE to the receptor for AGE (RAGE). This in turn leads to intracellular signal transduction, the activation of NF-κB, which then translocates to the nucleus and modulates gene expression as a transcription factor. RAGE can also regulate TNF-α expression. Other receptors such as lipoprotein receptor 1 (LOX-1) can also activate NF-κB by increasing production of intracellular reactive oxygen or reducing nitric oxide within the cell [48]. AGEs may also be linked to the development of chronic diseases associated with inflammatory processes such as atherosclerosis, cancer, Alzheimer’s disease, or diabetic retinopathy [49]. Moreover, an increase in insulin production caused by hyperglycemia may increase the endogen production of AA, as insulin influences the rate limiting enzymes responsible for the conversion of LA to AA, thereby promoting an inflammatory effect [50].

#### 3.1.4. Polyphenols

Polyphenols are a heterogeneous group of bioactive substances that are found in plant-based foods. Termed secondary phytochemicals, they can be subdivided into flavonoids, lignans, stilbenes, and phenolic acids, and are known to have a wide spectrum of benefits on health including antioxidant and anti-inflammatory effects [30,51,52]. Antioxidant properties are attributed to their ability to scavenge a wide range of ROS and chelate metal ions. Furthermore, polyphenols interact with a range of pathways (ex. NF-κB or MAPK) and have modulatory effects on cyclooxygenases (COXs), which decreases inflammation [51]. This anti-inflammatory potential was demonstrated in recent clinical trials where polyphenols reduced inflammatory markers such as TNF-α or IL-6 in elderly adults, with and without metabolic syndrome [53,54]. Additionally, polyphenols have a regulatory effect on the gut microbiota. Around 90% are not absorbed in the small intestine but rather transported to the large intestine where they are metabolized by microbes into metabolites such as SCFA. In addition, polyphenols may also have a beneficial effect on the composition of the microbiota, promoting growth of beneficial bacteria such as lactobacillus [55].

### 3.2. Anti-Inflammatory Potential of Dietary Patterns

Due to the complex interactions of different nutrients within a particular diet, the focus has shifted towards research on the effects of dietary patterns instead of single nutrients [56]. Several inflammatory scores have been developed to quantify the inflammatory potential of a diet, such as the DII and the Empirical Dietary Inflammatory Index (EDII). While the DII calculates the inflammatory potential of diet using single components such as spices or fatty acids [39], the EDII targets food groups such as processed meats or leafy green vegetables (Table 1) [57]. Both use a scoring system attributing a specific value to different food groups or components, depending on their inflammatory potential. These values are summarized according to the respective diet, generating a score representing inflammatory potential. Higher scores indicate a higher pro-inflammatory potential and are associated with higher inflammatory markers. The most extensively examined dietary pattern in terms of its association with inflammation is the Mediterranean diet (MD), characterized by a high intake of vegetables, legumes, fruits, olive oil, fish, and grains [58]. Plant-based dietary patterns such as the MD or the DASH (Dietary Approaches to Stop Hypertension) have been shown to be inversely correlated to inflammatory and oxidative markers. A high adherence to MD is associated with a decrease in CRP, IL-6, TNF-α, as well as biomarkers indicating oxidative stress such as ox-LDL, 8-OH-dG, and others. Simultaneously, there was a positive correlation for an increase in markers associated with radical oxygen species (ROS) detoxification [56,59]. Another dietary pattern studied for its anti-inflammatory potential is the ketogenic diet. Its main characteristic is the limitation of carbohydrates to 20–50 g per day, forcing the body into a ketogenic state where ketone bodies are produced by oxidizing fatty acids to form a source of energy [60]. Growing evidence of this diet’s anti-inflammatory properties highlight various mechanisms including a shift in the gut microbiota causing increases in folate production, inhibited assembly of certain inflammasomes, and/or activation of a specific G-Protein coupled receptor expressed on several immune cells [60,61]. Similarly, adherence to a Paleolithic diet, rich in plant-based and non-processed animal products but avoiding grain-based and processed foods, added sugar, salt, and dairy, has also been linked to a decrease in inflammation markers, especially CRP and oxidative biomarkers [62,63]. The consistent anti-inflammatory effects of plant-rich diets may be explained by the high content of anti-inflammatory nutrients mentioned above: ex. certain PUFAs, fibers, complex carbohydrates, and polyphenols. In contrast, there are multiple trials linking the “Western dietary pattern”, rich in processed meats, refined grains, or sugary beverages to an increase in inflammatory markers [59,64]. These results largely correspond with patterns in the EDII and the DII [65]. However, some EDII findings are highly controversial and counterintuitive, including a suggested pro-inflammatory effect of fish despite its high (anti-inflammatory) n-3 FA content. Findings of the epidemiological-based EDII might have been triggered by the food preparation methods (e.g., deep-frying). Similarly, a marginal pro-inflammatory effect of tomato-based products has been reported in a recent meta-analysis, but is not yet confirmed [66].

### 3.3. Immunonutrition

Anti-inflammatory or immune-modulating nutrients have already been applied in immunonutrition, which has the potential to influence immune system activity. There is no standard immunonutrition in terms of nutrients included and their concentrations. However, the formulae all combine several nutrients rather than single ones, including n-3 FA, vitamin D, selenium, nucleotides, and sulfur-containing amino acids or glutamine and arginine, which are given in supranormal dosages to induce a pharmacological effect [67,68,69,70]. Immunonutrition or immune-enhanced nutrition has become a topic of interest particularly in oncology, and in surgical or critically ill patients. Nevertheless, despite the identified anti-inflammatory properties, the use of immune-enhancing nutrition products in research and clinical practice produces conflicting results depending on the patient population. Moreover, the composition, amount, and timing are still under discussion, which also possibly contribute to the varying findings [71]. A recent meta-analysis of usage in esophageal cancer patients undergoing esophagectomy was unable to show a benefit on post-op infection rates compared to standard nutrition [72]. For head and neck cancer patients undergoing surgery, a Cochrane review found no improvement in length of stay or post-operative infection, but a possible benefit regarding fistula formation. Overall, the levels of evidence, however, were low [73]. Another systematic review by Yang et al. [74] showed that immunonutrition might decrease post-operative wound infections and shorten the length of hospital stay in patients undergoing surgery for pancreatic cancer. These results are in line with a comprehensive meta-analysis from 2020, which included 5983 cancer patients with surgery in 61 RCTs and reported positive effects on several clinical outcomes. Significant benefits on sepsis or all-cause mortality were not detected [70]. Correspondingly, ESPEN currently recommends the use of immunonutrition especially for upper GI cancer patients undergoing surgical treatment [75], or for malnourished cancer patients undergoing major surgery [76]. ESPEN guidelines for critical care [77] only recommend glutamine with a possible supplement of n-3 FA in burn and trauma cases, while the German Association for Clinical Nutrition (DGEM) [78] recommends excluding immune-modulating enteral nutrition and only advises restricted use in parenteral nutrition. In conclusion, the current evidence for beneficial effect of immunonutrition is still inconsistent and largely depends on the patient population.

## 4. Is Nutrition a Foe? How Inflammation Influences Response to Therapy

Although growing numbers of trials increase and strengthen knowledge on the anti-inflammatory effects of nutrients and dietary patterns, nutrition seems to have an opposite influence in inflammatory malnutrition. If DRM occurs with systemic inflammation, inflammatory mechanisms further aggravate malnutrition. When planning a nutritional intervention, it is crucial to classify a patient’s malnutrition based on etiology, even though it can be challenging in clinical practice [1]. Results of clinical trials support ESPEN and ASPEN classifications of malnutrition, showing differences in biomarker levels in acute versus chronic malnutrition and thus suggesting differences in pathophysiological pathways activated in each of these categories. The different pathophysiological pathways might also explain why varying types of malnutrition respond differently to nutritional support [79]. For nutritional therapy to be an effective integral part of a treatment plan, a “one fits all” approach has proven insufficient. Rather, influencing factors, underlying mechanisms, and biological parameters for (non)response should be identified and further explored in order to improve and individualize nutritional management [18,80].

### 4.1. Research on Predictors for Treatment Response

Evidence of improved clinical outcomes following nutritional therapy in malnourished patients has been strengthened by recent high-quality RCTs [20]. However, though overall medical inpatient populations with malnutrition have been shown to benefit from nutritional treatment, the heterogeneity of study populations and interventions produced contradictory findings in the past [6,17,20,81,82]. Not every patient population reacts to nutritional therapy in the same way, as nutritional and metabolic needs seem to differ. Even within a relatively homogenous group, response to nutritional therapy can vary depending on factors such as malnutrition severity [83,84] or kind of disease [25,85,86,87,88], as seen in secondary analyses of trials including EFFORT. One particular meta-analysis showed stronger beneficial effects of nutritional therapy in patients with established malnutrition compared to those only at nutritional risk [20]. In the search for predictors, inflammation is another identified factor that influences responses to nutritional therapy.

### 4.2. Inflammation as a Predictor for Treatment Response

The persistent catabolism during inflammation leads to loss of muscle mass if muscle proteolysis exceeds muscle protein synthesis. While nutritional support can potentially reverse this imbalance [20], in highly inflamed patients, the catabolic process seems to be irreversible, even under nutritional support [89]. This can result in nonresponse [90,91] or even harmful effects due to a possible overfeeding [5,91,92,93,94,95,96,97]. Constant and extensive systemic inflammation in acutely malnourished ICU and surgical patients [6] is the considered main reason for the nonresponse to nutritional therapy mentioned above [5]. Similarly, acute malnutrition in medical inpatients was associated with changes in biomarkers, which reflect inflammatory or infectious processes [79]. When seeking an explanation for the conflicting results concerning the effect of nutritional therapy, it is thus important to consider ESPEN and ASPEN’s proposed etiologic classification of malnutrition, which distinguishes between DRM with or without inflammation and malnutrition due to acute or chronic disease, respectively.

Indeed, the response to nutritional therapy depends on the inflammatory status. As an acute-phase protein, CRP is one of the most popular biomarkers for inflammation. The production of acute-phase proteins in the liver is induced by pro-inflammatory cytokines [98]. CRP levels of >100 mg/L (an indication of high inflammation) have been shown to be associated with a lower treatment response in malnourished medical inpatients who did not benefit significantly from nutritional therapy in EFFORT. However, 30-day mortality in patients with low and moderate CRP levels was significantly reduced (OR 0.34 and 0.41, respectively) [7]. Similarly, comparing CRP levels in the cancer patients subgroup, those with CRP > 100 mg/L showed no response to treatment, suggesting that inflammation is an important driver in addition to the main diagnosis [99]. These results are in line with former trials, e.g., by Gariballa and Forster, in which acute-phase response defined by CRP >10 mg/L had significant negative effects on nutritional status and clinical outcome [100].

Albumin is another acute-phase protein and established biomarker for inflammation. It is also associated with inflammatory DRM, as low levels in acute illness are mainly caused by inflammation and it is a strong prognostic marker for mortality. In contrast to CRP, albumin, however, did not predict the response to nutritional therapy within the EFFORT cohort. Stratifying by CRP, only low and moderately inflamed patients responded while highly inflamed did not. Additional stratification by albumin concentration added no further informational value. Albumin can therefore not be considered a suitable inflammatory marker for response to nutritional therapy [98,101].

### 4.3. Explanatory Approaches for Non-Response in Highly Inflamed Patients

Inflammation, reflected by elevated CRP and decreased albumin levels, may at least partly explain nonresponse to nutritional therapy within highly inflamed patients such as the critically ill [8]. The influence of unbalanced autophagy has been discussed as a contributing factor, as its balance is reported to be crucial in the inflammatory response [102]. This cellular self-degradative process is induced by stressors (including underfeeding) and is an essential adaptation mechanism for cell detoxification during acute disease and inflammation [95,103]. Meanwhile, the products of this breakdown are reused in cellular metabolism and serve as an energy source during starvation. Food intake is a well-known suppressor of autophagy [95], leading to an “inadequate clearance of cell damage and microorganisms” [104]. In critically ill patients, overfeeding by excessive nutrition during acute phase [105] has been shown to impair autophagy [106]. On the other hand, He et al. suggested that overfeeding could also induce autophagy, leading to unbalanced “over-autophagy”, with subsequent excessive cellular breakdown and cell death—again highlighting the importance of a balanced autophagy [107]. As a consequence, disease-related anorexia and subsequent downregulation of nutritional intake by cytokines may have physiological benefits on this process and clinical outcome [95,104]. However, when severity of illness increases, autophagy can become excessive, resulting in a pathological mechanism and increased cell death. This implies that autophagy in critical illness is not beneficial or harmful per se, but implies that adequate nutritional approaches should be taken into consideration to achieve a balanced autophagy [108].

Another possible explanation for nonresponse is the higher prevalence of refeeding syndrome and the use of PN in highly inflamed, severely malnourished patients, which can result in higher complication rates and potentially lead to contrary outcomes [92,95,109]. Lastly, feeding via continuous PN or EN may blunt protein synthesis and thus contribute to the imbalance in muscle protein degradation and synthesis [5,91].

## 5. Clinical Practice—Nutritional Therapy for Severely Inflamed Patients

ICU patients are at risk for malnutrition and still require nutritional therapy to prevent impaired recovery from critical illness [77]. Clearly, the phase and severity of the acute illness and inflammatory response play important roles in terms of nutritional needs and response to nutritional therapy. Although it can be challenging in clinical practice, a consistent adaptation of nutritional management during the course of critical illness is crucial [92,108,110,111].

Despite growing knowledge about connections between inflammation, malnutrition, and low response to nutritional therapy in highly inflamed patients, a satisfactory approach is still unclear. Nutritional strategies remain controversial, including questions about timing, amount, and routes of administration. Several trials have been conducted to resolve conflicting recommendations by medical guidelines on nutritional therapy in highly inflamed patients [89,96,97,100,101,105,112]. Some suggested withholding nutritional therapy until systemic inflammation has subsided. For instance, the EPaNIC trial, including approximately 5000 patients, compared late initiation of additional PN (recommended by American and Canadian guidelines) in patients not reaching caloric targets with EN to early substitution (ESPEN guideline) [92]. Despite higher acute inflammation levels and incidence of hypoglycemia, overall late initiation was superior, resulting in shorter length of hospital and ICU stay, and reduced mechanical ventilation and renal replacement therapy compared to early initiation. Rates of ICU infections and cholestasis, as well as health care costs were also lower. In contrast, Patel et al. pointed out that the effect of autophagy depends on the stages of disease, as mentioned above. Withholding nutritional therapy to preserve autophagy might only be beneficial in an early phase in mild critical illness, while not delaying nutritional therapy for as long as multiple days [108]. Meanwhile, the PermiT trial demonstrated that underfeeding while upholding protein targets in the critically ill did not improve survival [113]. However, nutritional support has also been reported to decrease complication rates in critically ill, undernourished patients [94,114].

As ICU patients are typically highly inflamed, research on the interaction between inflammation, nutritional status, and response to therapy is often focused on critical care. However, pathophysiological mechanisms in other highly inflamed patients are similar and it is plausible that proposed explanations for nonresponse and approaches for nutritional therapy also apply to them. This might also be the case in the medical inpatient cohort in EFFORT [7]. The high heterogeneity of this patient group (comorbidities and state of disease) still requires incorporation of their subsequent individual nutritional needs into future nutritional strategies [69].

## 6. Personalized Nutrition

Despite recent promising results and the possibility of stratifying patients, the implications for clinical practice must still be determined [1,5]. Current evidence suggests that specific nutritional needs are not only based on traditional parameters such as body weight, sex, and age for calculating caloric targets, but also on illness-specific (e.g., comorbidities and acute vs. chronic course) or other patient-specific factors (e.g., genetic traits or metabolomic markers), including inflammatory status [115,116]. Identifying underlying factors, mechanisms, and biological parameters and using them to improve the quality of nutritional therapy will help adapt ideally to individual patients’ needs.

However, identification and stratification do not mean withholding nutritional therapy from non-responders, such as patients with CRP > 100 mg/L. Rather, they further emphasize the need for thorough evaluation of the underlying causes, followed by an appropriate, individualized treatment. Even though it seems counter-intuitive, this corresponding treatment may, for instance, involve withholding nutritional treatment during acute-phase response in ICU patients. However, more research is needed on how to feed highly inflamed malnourished patients [1,20].

The concept of precision or personalized nutrition takes into account that not all patients show the same response to an intervention, and that it is necessary to provide them with “personalized” nutritional therapy based on their individual condition and requirements. Following the identification of relevant factors and associated biomarkers, patients may be stratified into subgroups according to their presumed response to nutritional therapy [1,117]. In addition to inflammation represented by CRP, other biomarkers have been found to be associated with DRM, including procalcitonin, proadrenomedullin, and copeptin [79]. Other biomarkers also predict response to nutritional support such as handgrip strength [118], sarcopenia [1,119], kidney function by estimated glomerular filtration rates (eGFR) [88], or triiodothyronine (T3) serum concentration [25]. Nutritional interventions may be adapted based on these findings. If patients, e.g., with low handgrip strength, do not respond to the nutritional intervention, they may require a specialized protocol or nutrient composition to fulfill their individual needs.

Once established, recommendations for evidence-based personalized nutrition may enable clinicians to treat all patients effectively, including those who do not benefit or are even harmed by “traditional” interventions.

## 7. Conclusions

There is increasing evidence highlighting that inflammation and nutrition are strongly linked: nutrition influences the body’s inflammatory reaction, and inflammation influences the effects of nutrition on many different levels. Research suggests that patients with high inflammation—including cancer patients—may not benefit from current nutritional treatment plans, and optimal approaches for the use of nutritional therapy in highly inflamed patients are still inconsistently understood. Based on the existing literature, it is reasonable, however, to stratify malnourished patients according to inflammatory status and anticipated response to the therapy. In the future, more personalized nutritional approaches must be developed to specify type, amount, composition, and timing. These adaptations represent important steps toward individualized nutritional management, and the provision of effective nutritional therapy to highly inflamed patients who do not respond to existing nutritional strategies. Future research must focus on this highly vulnerable group of patients at high risk for malnutrition-related adverse clinical outcomes, and then interventions should be applied to determine the best clinical approach.

## Figures and Tables

**Table 1:** Overview of selected pro-inflammatory and anti-inflammatory food components according to the Dietary Inflammatory Index (DII) [39] and food groups according to the Empirical Dietary Inflammatory Index (EDII) [57].

Pro-Inflammatory		Anti-Inflammatory	
Food Components	Food Groups	Food Components	Food Groups
Saturated fatty acids	Refined grains	Flavonoids	Coffee
Trans fatty acids	Carbonated Beverages	Fibre	Wine
Cholesterol	Meat, especially red or processed	Poly-unsaturated fatty acids	Beer
Vitamin B12	Organ meat	Omega-3 fatty acids	Fruit juice
Iron		Omega-6 fatty acids	Tea
		Turmeric, garlic, ginger	Leafy green vegetables
		Vitamin A, D and E, β-Carotene	Dark yellow vegetables
		Vitamin C, B6, niacin	
		Magnesium, zinc	
